# C1q acts in the tumour microenvironment as a cancer-promoting factor independently of complement activation

**DOI:** 10.1038/ncomms10346

**Published:** 2016-02-01

**Authors:** Roberta Bulla, Claudio Tripodo, Damiano Rami, Guang Sheng Ling, Chiara Agostinis, Carla Guarnotta, Sonia Zorzet, Paolo Durigutto, Marina Botto, Francesco Tedesco

**Affiliations:** 1 Department of Life Sciences, University of Trieste, Trieste 34127, Italy; 2 Department of Human Pathology, University of Palermo, Palermo 90127, Italy; 3 Centre for Complement and Inflammation Research, Department of Medicine, Imperial College, London W12 0NN, UK; 4 Institute for Maternal and Child Health, Istituto di Ricovero e Cura a Carattere Scientifico Burlo Garofolo, Trieste 34137, Italy; 5 Istituto di Ricovero e Cura a Carattere Scientifico, Istituto Auxologico Italiano, Milan 20145, Italy

## Abstract

Complement C1q is the activator of the classical pathway. However, it is now recognized that C1q can exert functions unrelated to complement activation. Here we show that C1q, but not C4, is expressed in the stroma and vascular endothelium of several human malignant tumours. Compared with wild-type (WT) or C3- or C5-deficient mice, C1q-deficient (*C1qa*^−/−^) mice bearing a syngeneic B16 melanoma exhibit a slower tumour growth and prolonged survival. This effect is not attributable to differences in the tumour-infiltrating immune cells. Tumours developing in WT mice display early deposition of C1q, higher vascular density and an increase in the number of lung metastases compared with *C1qa*^−/−^ mice. Bone marrow (BM) chimeras between *C1qa*^−/−^ and WT mice identify non-BM-derived cells as the main local source of C1q that can promote cancer cell adhesion, migration and proliferation. Together these findings support a role for locally synthesized C1q in promoting tumour growth.

## Abstract (web summary)

C1q is known to initiate the activation of the complement classical pathway. Here, the authors show the C1q is expressed in the tumour microenvironment and can promote cancer cell migration and adhesion in a complement activation-independent manner.

## Graphical abstract

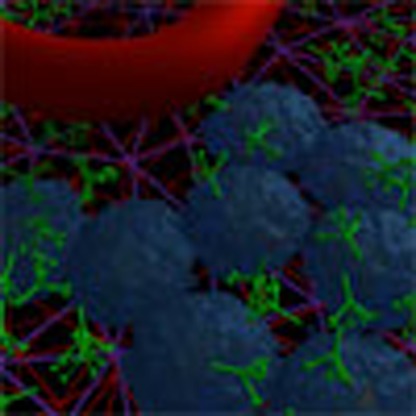

Cells undergoing malignant transformation acquire the ability to proliferate in an uncontrolled manner invading the surrounding tissue and spreading to distant organs via the circulatory or lymphatic systems^1^. There is strong evidence that tumour growth not only depends on the accumulation of genetic abnormalities in the originating cancer cells, but also on the local microenvironment that can provide a permissive niche for the survival, growth and migration of the cancer cells^2^,^3^. Tumour cells recognized by the immune system are kept under surveillance by the combined action of the innate and adaptive immune systems and are either eliminated or remain quiescent for long time^4^,^5^. The latter situation is difficult to detect *in vivo* due to the absence of clinical symptoms, but its existence is supported by the findings of dormant tumours at autopsy of patients deceased for other reasons^6^,^7^,^8^. Conversely, fast growing cancer cells develop strategies to escape immune defences^5^,^9^,^10^,^11^. They can trigger local inflammation that promotes disease progression rather than controlling tumour growth^12^,^13^,^14^. This process is characterized by the peri-tumoral recruitment of leucocytes with immunosuppressive properties such as tumour-associated macrophages, myeloid-derived suppressor cells (MDSCs) and regulatory T cells^15^.

The complement (C) system, a well-known arm of innate immunity^16^,^17^, is one of the immune players present in the tumour microenvironment as suggested by the finding of C deposits on tumour tissue from patients with breast, papillary thyroid, colorectal and ovarian carcinoma^18^,^19^,^20^. The involvement of C in cancer immunosurveillance has long been neglected until monoclonal antibodies (mAbs) to tumour-associated antigens were introduced in cancer therapy^21^. In addition to mediating antibody-dependent cell cytotoxicity (ADCC), some mAbs can trigger C activation that helps control tumour growth by a direct cytotoxic effect on cancer cells and/or by promoting inflammation^22^,^23^,^24^,^25^. The advantage of the C system over ADCC is that it is made of soluble components readily available at tissue sites where they are secreted by local and recruited cells and sometimes by the same tumour cells. However, the contribution of C to the killing of cancer cells remains unclear because tumour cells overexpress membrane-bound C regulatory molecules (CRPs) such as CD46, CD55 and CD59 (refs 24, 26, 27) that can limit the cytotoxic effects of C activation. The importance of CRPs in tumour protection has been highlighted by a recent study showing that bispecific antibodies containing C-fixing anti-CD20 mAb and neutralizing Abs to CRPs are highly effective *in vivo* in cancer immunosurveillance^28^. Furthermore, data accumulated over the last few years suggest a tumour-promoting role for the C system^29^. Markiewski *et al.*^30^ first reported that C5a, generated in the tumour microenvironment as a result of C activation, favours cervical cancer growth by recruiting and activating MDSCs. This observation together with similar findings in mouse models of lung and ovarian cancers^31^,^32^, and more recently of mesenchymal and epithelial carcinogenesis^33^, challenges the concept that C activation at tumour site is always beneficial for cancer patients. However, the clinical relevance of these findings in animal models remains to be established especially as C5a has also been found to control tumour growth^34^,^35^ and to exert opposite effects depending on the amount available in the tumour microenvironment^36^. Irrespective of the beneficial or harmful impact of C on tumour growth, all the C-mediated effects have been found to be associated with fragments such as C3a and C5a implying C activation^37^,^38^.

Here we present data showing that the first component of the C system, C1q, can act as tumour-promoting factor by favouring adhesion, migration and proliferation of cancer cells as well as angiogenesis and metastasis. C1q is synthesized in the tumour microenvironment and acts like a protein of the extracellular matrix favouring tumour growth and metastasis. These findings demonstrate that C1q can contribute to tumour progression and invasion regardless of C activation.

## Results

### C1q is expressed in the microenvironment of human cancers

**Figure 1:**
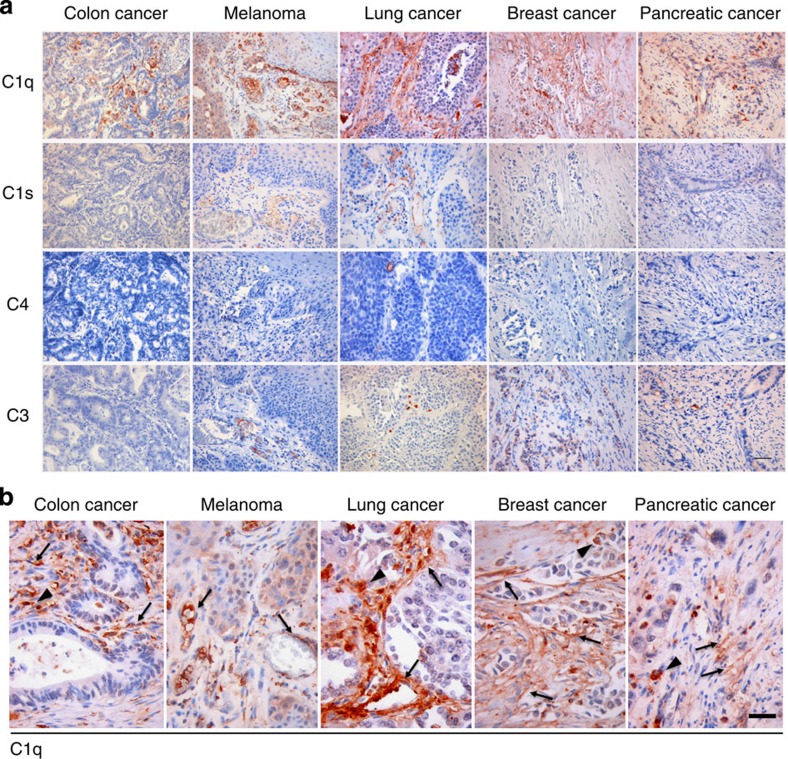
Immunohistochemical analysis of classical C components in human tumours. (**a**) Representative microphotographs showing the expression of C1q, C1s, C4 and C3 in different malignant cancer histotypes. Streptavidin–biotin–peroxidase complex system with AEC (red) chromogen; scale bars, 100 μm. (**b**) C1q expression in tumour-associated stroma of different cancer histotypes. Highlighted are monocytoid cells suggestive of tumour-infiltrating myeloid elements (arrow heads) and mesenchymal elements including vascular endothelial cells and spindle-shaped fibroblasts (arrows). Streptavidin–biotin–peroxidase complex system with AEC (red) chromogen; scale bars, 50 μm.

**Figure 2:**
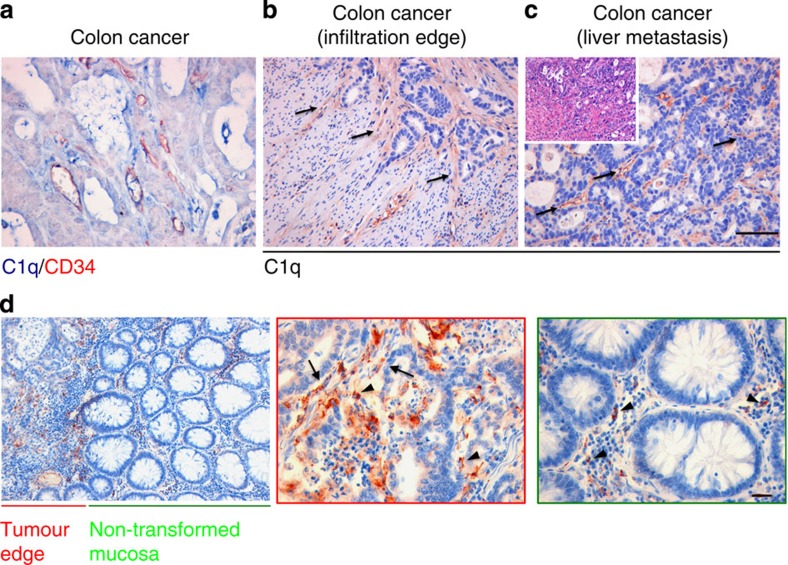
Immunohistochemical analysis of primary and metastatic colon carcinoma for deposition of C1q. (**a**) Expression of C1q on vascular endothelia demonstrated by double-marker immunohistochemical analysis showing co-localization of C1q (blue signal) and CD34 (red signal) in vessels; scale bars, 50 μm. (**b**) C1q expression in stromal tissue neighbouring neoplastic glandular foci at the edge of tumour infiltration in colon adenocarcinoma (arrows); scale bars, 100 μm. (**c**) C1q expression in the tumour-associated stroma at sites of liver metastasis of colon adenocarcinoma. Streptavidin–biotin–peroxidase complex system with AEC (red) chromogen; scale bars, 100 μm. (**d**) Representative microphotographs showing differential C1q expression in the stroma of cancer-involved and non-involved mucosa at lower magnification (left panel, scale bar, 200 μm) and higher magnification (middle and right panels, scale bars, 50 μm). C1q-expressing, tumour-infiltrating myeloid elements (arrow heads) and mesenchymal elements including vascular endothelial cells and spindle-shaped fibroblasts (arrows) are differently enriched in the two conditions. Streptavidin–biotin–peroxidase complex system with AEC (red) chromogen.

We have recently reported that C1q favours trophoblast invasion of maternal decidua^39^ and promotes angiogenesis in wound healing^40^. As these features are shared to some degree by growing tumours, the possibility that C1q may contribute to this process was raised. We initially searched for the presence of C1q, C1s, C4 and C3 in a panel of 30 invasive malignant neoplasm specimens including 6 cases of colon adenocarcinoma, 6 cases of melanoma, 6 cases of lung adenocarcinoma, 6 cases of breast adenocarcinoma and 6 cases of pancreatic adenocarcinoma. As shown in Fig. 1, a strong signal for C1q was detected in all tumour specimens examined, while other C components were either absent (C4) or mildly expressed (C1s and C3) suggesting that C1q deposition did not result in the activation of the classical pathway. Within the tumour microenvironment, C1q was mainly expressed by mesenchymal elements including vascular endothelial cells and spindle-shaped fibroblasts (arrows) and monocytoid cells suggestive of tumour-infiltrating myeloid elements (arrow heads) (Fig. 1b). C1q expression within newly formed tumour-associated vascular endothelia was further confirmed by double-staining with CD34, an endothelial marker (Fig. 2a). Similarly, the C1q expression in mesenchymal and immune cells was corroborated by double-staining immunofluorescence analyses of invasive colon and breast cancer specimens using markers specific for epithelial (pan-cytokeratin), mesenchymal (vimentin) or immune cells (CD45) (Supplementary Fig. 1). In agreement with the immunohistochemical analyses, malignant cells, identified by cytokeratin immunostaining (Supplementary Fig. 1, left panels, green signal), did not show C1q expression (red signal). By contrast, mesenchymal elements, detected using vimentin as a marker (middle panels, green signal), diffusely expressed C1q (red signal). Immune cells positive for CD45 (right panels, green signal) also expressed C1q (red signal), though this was not visible in all infiltrating cells. Notably, C1q expression in the mesenchymal elements appeared to be associated with cancer invasion since C1q was clearly detected in stromal elements at the tumour invasion edge (Fig. 2b), but not in the tumour cells (Supplementary Fig. 2). Consistent with this, C1q was not detected in non-transformed and non-infiltrated peri-tumoral tissues (Fig. 2d). Moreover, the association between C1q expression and tumour stroma was not confined to primary lesions as it was also observed in the stroma of metastatic tumour foci (Fig. 2c). Overall, these *in situ* analyses confirmed that C1q expression within the tumour microenvironment is mainly limited to the stromal elements suggesting its relevance in cancer cell-extrinsic dynamics.

### Prolonged survival and reduced tumour mass in *C1qa* ^−/−^ mice

**Figure 3:**
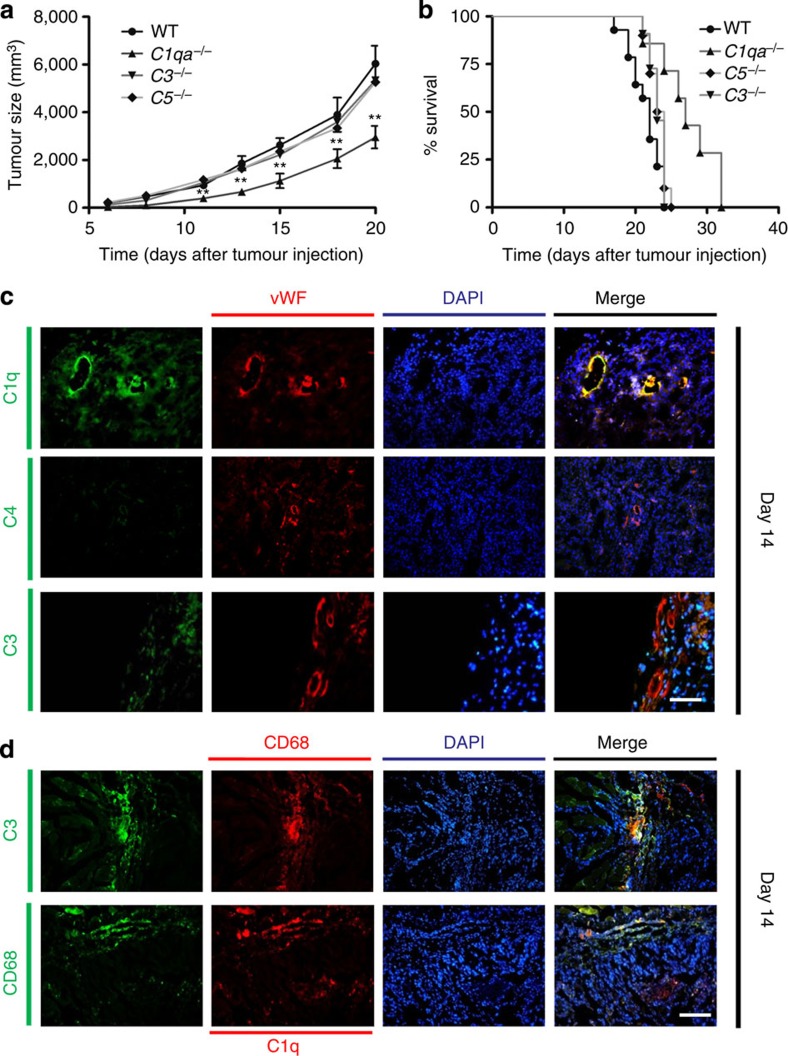
Effect of C1q on tumour growth and survival and analysis of tumour tissue for C deposition. WT (*n*=14), *C1qa*^−/−^ (*n*=7), *C3*^−/−^ (*n*=11) and *C5*^−/−^ (*n*=10) mice received an intramuscular injection of 2 × 10^6^ B16/F10 melanoma cells and the tumour size (**a**) and survival (**b**) was monitored at different time points. Pooled data from two independent experiments. Tumour size data are presented as mean±s.e.m. **P<0.01 (Two-way ANOVA, Tukey–Kramer test). Survival curve was analysed by Kaplan–Meier test. (*C1qa*^−/−^ versus WT: *P*<0.01; *C1qa*^−/−^ versus *C3*^−/−^: *P*<0.01; *C1qa*^−/−^ versus *C5*^−/−^: *P*<0.01; *C3*^−/−^ or *C5*^−/−^ versus WT: not significant) (**c**) Immunofluorescence analysis of tumour tissue for the distribution of C1q, C4 and C3. Melanoma tissue was double stained with fluorescent-labelled antibodies to C1q, C4 or C3 (green) and to vWF (red). The cell nuclei were stained with DAPI. Scale bars, 100 μm. (**d**) Immunofluorescence staining of tumour tissue for C1q and CD68+ cells. The tumour tissue was double stained with either fluorescent-labelled antibodies against C3 (green, 1:500) and CD68 (red, 1:200) or antibodies against C1q (red, 1:200) and CD68 (green, 1:200). The cell nuclei were stained with DAPI. Scale bars, 100 μm.

The presence in tumour tissues of C1q in the absence of other components of the classical C pathway led us to evaluate the contribution of this molecule to tumour growth in a syngeneic model of melanoma developed in C57BL/6 WT mice. After pilot experiments to define the optimal experimental conditions for inducing tumour growth and lung metastasis in a high proportion of mice, we selected the model in which 2 × 10^6^ B16/F10 melanoma cells are injected intramuscularly. In WT animals, the tumour mass reached a mean of 6 cm^3^ 20 days after the cell injection (Fig. 3a) and the mice were all humanely culled by 32 days (Fig. 3b). In contrast, *C1qa*^−/−^ mice exhibited a prolonged survival with significantly slower tumour growth throughout the period of observation, while the tumour growth and the survival rates of both C3- and C5-deficient animals did not differ from those of WT animals (Fig. 3a,b). A marked difference in the tumour growth rate between *C1qa*^−/−^and WT mice was also observed using the syngeneic mouse model of lung carcinoma (Supplementary Fig. 3). Consistent with the findings in human specimens, immunohistochemical analysis of the tumour masses showed marked deposition of C1q on vascular endothelium and stroma in the absence of C4. The C3 staining was scattered and mainly associated with tumour-infiltrating cells (Fig. 3c). To characterize better the cellular source of C1q and C3, the tumour sections were also stained with an antibody against CD68, a widely used marker for tissue macrophages. As shown in Fig. 3d, C3 was expressed almost exclusively in CD68^+^ cells, while C1q staining was present in both CD68^+^ and CD68^−^ cells, the latter most likely representing stromal cells. Moreover, kinetic analysis of C expression in the growing tumour revealed the presence of C1q as early as 2 days after tumour cell injection preceding the expression of C3 that was detectable only 2 days later and became obvious on day 6 (Supplementary Fig. 4a). Further analysis of C3 expression using an antibody that recognizes only C3 activation products failed to detect cleaved C3 fragments within the tumour sections (Supplementary Fig. 4b).

In subsequent experiments, we killed the animals at day 12 and analysed by flow cytometry the tumour-infiltrating immune cells. Although the tumour sizes were markedly different (Supplementary Fig. 5), the percentages and phenotype of CD4^+^ and CD8^+^T cells within the tumour mass were similar (Supplementary Fig. 6). In addition, we failed to detect a higher frequency or an increased suppressive ability of the myeloid-derived suppressor cells in WT mice that could explain the enhanced tumour growth in these animals compared with the *C1qa*^−/−^ mice (Supplementary Fig. 7a–c).

### Tumour growth in BM transplanted mice

**Figure 4:**
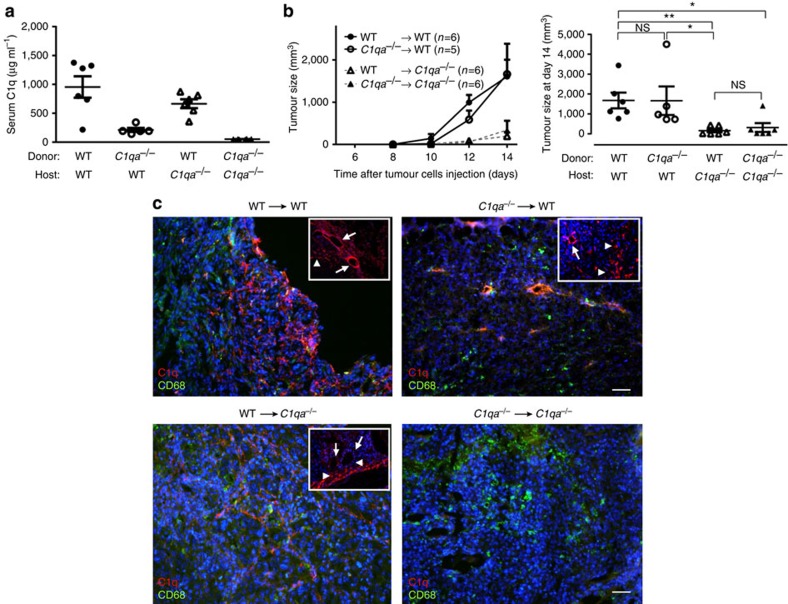
Tumour growth in BM transplanted mice. (**a**) C1q antigenic levels in reconstituted mice. Levels were measured by ELISA as described in the Methods section 2 months after BM transplant. Results are expressed in μg ml^−1^, referring to a standard curve derived from a known concentration of purified C1q. (**b**) Tumour mass measured on various days after tumour cell injection (left graph) and on end-point (day 14) when mice were culled and tumours were removed (right graph). Irradiated recipient mice are represented by a circle (WT) or a triangle (*C1qa*^−/−^). Data are shown as means±s.e.m. Each symbol represents an individual mouse; *P<0.05, **P<0.01, NS, not significant (two-way ANOVA). (**c**) Representative imagines showing staining for C1q (red) and CD68 (green) in tumour sections of BM transplanted mice at day 14; scale bars, 100 μm. Insets show details of cells (arrowheads) and vessels (arrows) stained for C1q; scale bars, 100 μm.

To determine the contribution of stromal-derived C1q to the development of the tumour, we generated reciprocal radiation bone marrow (BM) chimeras between *C1qa*^−/−^ and WT mice. In agreement with our previous findings^41^,^42^, 2 months after the BM transplant, C1q levels in the C1q-deficient recipients were almost comparable to those in control WT chimeras (Fig. 4a). In contrast, the C1q levels in WT mice reconstituted with *C1qa*^−/−^ BM cells were hardly detectable. The development of the tumour in the BM chimeras was then evaluated (Fig. 4b). A progressive increase in tumour was observed in all experimental groups during the period of observation. However, at each time point this increase was markedly less pronounced in the *C1qa*^−/−^ irradiated recipient mice regardless of the circulating C1q levels. Syngeneic reconstituted *C1qa*^−/−^ mice and chimeric mice becoming C1q sufficient after BM transplantation developed smaller tumours. Conversely, WT mice reconstituted with *C1qa*^−/−^ BM cells and syngeneic reconstituted WT mice showed accelerated tumour growth (Fig. 4b). Taken together these data indicate that the non-BM-derived C1q was a critical factor for the tumour growth. The cellular source of C1q was then analysed by immunofluorescence using antibodies against C1q (red) and CD68 to identify macrophages (green) (Fig. 4c). As expected, C1q was undetectable in *C1qa*^−/−^ mice reconstituted with *C1qa*^−/−^ BM cells, while it was present in endothelial cells and in CD68^+^ cells in WT mice receiving WT BM cells. Vascular endothelium and CD68^−^ non-BM-derived cells expressed C1q in WT mice reconstituted with *C1qa*^−/−^ BM cells. Conversely, endothelial cells did not stain for C1q in *C1qa*^−/−^ recipients of WT BM cells that colonize the tumour mass with macrophages expressing C1q.

### Effects of C1q on tumour angiogenesis and metastases

**Figure 5:**
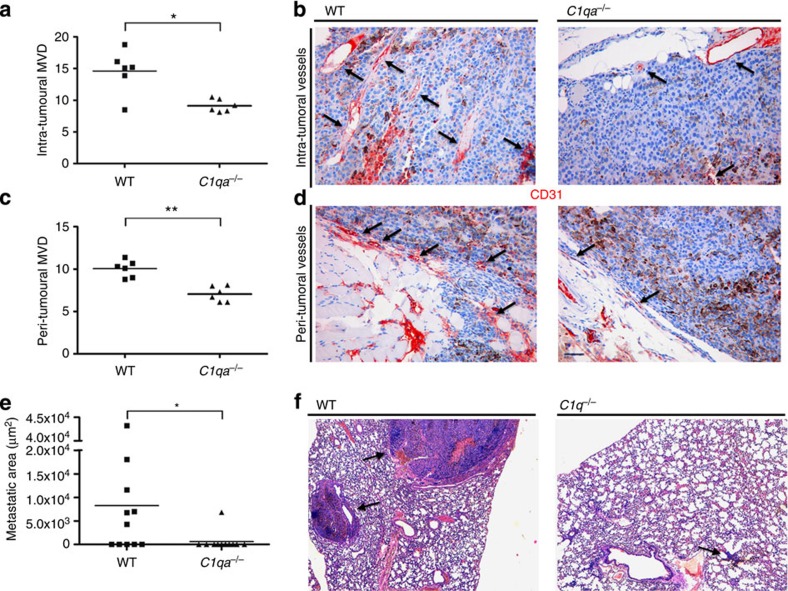
Angiogenesis and lung metastases in tumour-bearing mice. Quantification of intra-tumoral (**a**) and peri-tumoral (**c**) MVD on 10 different microscopic fields of tumour sections from 6 WT and 6 C1q-deficient mice immunostained for CD31. Pooled data from two independent experiments. Representative pictures of CD31-immunostained sections of tumour tissue from WT and C1q-deficient mice showing intra-tumoral (**b**) and peri-tumoral (**d**) CD31^+^ cells (arrows). Each symbol represents the number of vessels counted in one microscopic field (10 fields per mouse, scale bars, 100 μm) in 6 WT (filled square) and 6 *C1qa*^−/−^(filled triangles) mice. Horizontal lines indicate median. *P<0.05, **P<0.01 (Mann–Whitney *U*-test). (**e**) Measurement of metastatic areas in the lungs of tumour-bearing WT (*n*=11) and C1q-deficient mice (*n*=11). Each symbol represents the lung metastatic area (μm^2^) in each WT (filled squares) and *C1qa*^−/−^(filled triangles) mice. Pooled data from two independent experiments. Horizontal lines indicate median, *P<0.05 (Mann–Whitney *U*-test). Representative images showing metastases in both groups of mice (**f**). Scale bars, 100 μm.

As cancer development largely depends on angiogenesis^43^,^44^,^45^, we then examined the degree of new vessel formation in the tumour tissue. We found that the vascular density in tumour-bearing WT mice was significantly higher than in *C1qa*^−/−^ animals, particularly in the peri-tumoral area (Fig. 5a–d). We also searched for metastatic melanoma cells in the lungs, the organ preferentially colonized by the cancer cells that escape from the primary tumour site, and detected metastases in 6 out of 11 WT mice but only in 1 out of 11 *C1qa*^−/−^ mice (Fig. 5e–f).

### Biological effects of C1q on murine melanoma cells

**Figure 6:**
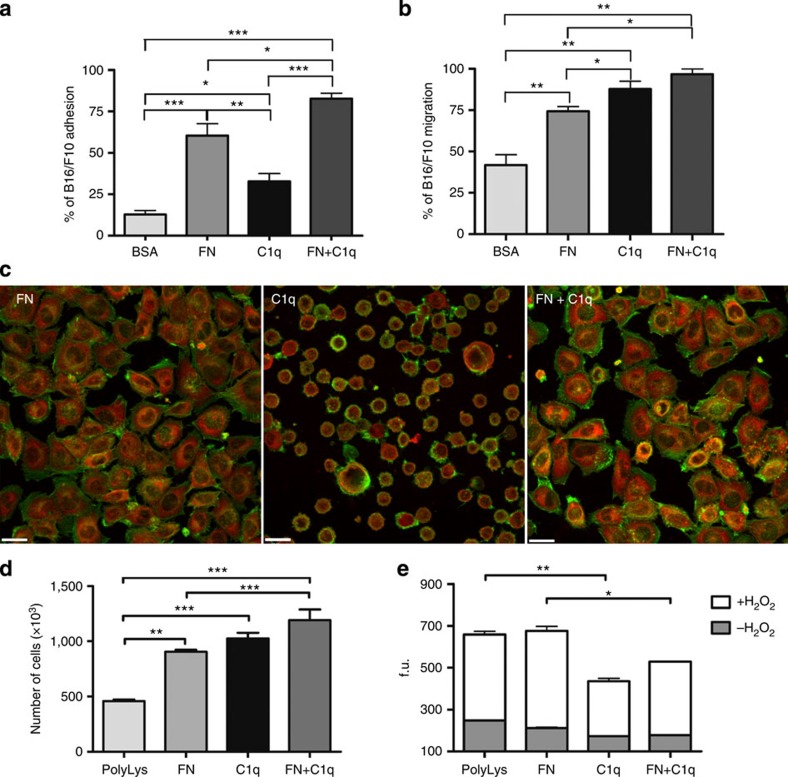
Effects of C1q on B16/F10 melanoma cell biology. Cell adhesion (**a**), migration (**b**), proliferation (**d**) and apoptosis (**e**) were performed as detailed in Methods section. Data from at least five independent experiments are presented as mean±s.e.m., *P<0.05, **P<0.01, ***P<0.005 (one-way ANOVA, Bonferroni corrections). (**c**) Representative images of tumour cells after incubation with C1q, FN and FN+C1q for 30 min. Cells were stained with NBD-phallacidin (green, dilution 1:50) and mouse monoclonal anti-paxillin Ab (dilution 1:100) followed by Cy3-conjugated goat anti-mouse IgG (red, dilution 1:300) and analysed by confocal microscopy. Scale bars, 23 μm. f.u., fluorescence units.

Having established that C1q is present in the tumour mass, we sought to investigate the mechanism(s) by which C1q contributed to tumour growth by analysing its ability to promote adhesion, proliferation and migration of cancer cells. To evaluate the C1q-mediated effects, melanoma cells were labelled with the fluorescent probe FAST DiI (Molecular Probes, Invitrogen) and allowed to adhere to plate-bound C1q or a mixture of fibronectin (FN) and C1q, using bovine serum albumin (BSA) and FN as negative and positive controls, respectively. C1q was able to promote the adhesion of 33% of cells as opposed to nearly 13% of cells adhering to BSA. In addition, it enhanced the pro-adhesive activity of FN to ∼80% following its interaction with this extracellular matrix protein (Fig. 6a). A high fraction of cells bound to FN appeared spread out and displayed actin-containing stress fibres, in contrast with the round morphology exhibited by the great majority of those attached to C1q and by a proportion of cells adhering to C1q bound to FN (Fig. 6c). The ability of C1q to induce cell migration was examined by adding the tumour cells to the upper chamber of a transwell system and allowing them to migrate through an insert coated with C1q or FN or both. C1q was slightly more effective than FN, allowing migration of 87% of cells that increased to 96% with the mixture of C1q and FN (Fig. 6b).

Given the *in vivo* findings that C1q promotes cancer progression, we then explored whether C1q might contribute to tumour growth by stimulating the proliferation of cancer cells. To this end, the melanoma cells were incubated with either plate-bound C1q or FN or the mixture of both and the number of proliferating cells was counted with the Coulter Particle Counter. As shown in Fig. 6d, C1q induced cell proliferation comparable to that obtained with FN and the total number of proliferating cells increased further after stimulation with both. In addition, cells adhering to C1q, unlike those bound to FN, were protected from apoptosis induced by oxidative stress (Fig. 6e). Moreover, a reduced frequency of proliferating tumour cells was also detected in the C1q-deficient mice *in vivo* using BrdU incorporation (Supplementary Fig. 7d).

## Discussion

During cancer development, the tumour microenvironment with infiltrating immune and non-immune cells, as well as the extracellular matrix undergoes substantial changes that can influence tumour progression^46^,^47^. The data presented in this study demonstrate that C1q contributes to these changes independently of C activation by acting as an external component of the extracellular matrix and favouring tumour growth and invasion.

Deposits of C components have been reported in different human tumours and have been interpreted as the result of C activation induced by several triggers including antibodies to tumour-associated antigens, immune complexes and cell damaged by necrosis and apoptosis^18^,^19^. The extent of C activation, that in some cases proceeds up to the assembly of the terminal complex^48^, depends on the tumour type and the degree of inflammation associated with tumour invasion. We found that C1q was the predominant C component deposited in all the tumours examined in this study. Its localization on endothelial cells and stroma is reminiscent of its distribution in human decidua, where it is locally synthesized and secreted by several cells including endothelial cells and trophoblasts^39^,^49^. Although C1q deposition is usually regarded as an indication of classical pathway activation, our failure to detect C4 makes this unlikely and rather suggested an alternative mechanism for the C1q involvement at tumour site, not necessarily related to classical pathway activation. However, we cannot exclude complement activation through the alternative pathway as suggested by the weak C3 staining observed with an antibody against human C3d.

Data accumulated in recent years have revealed non-canonical functions exerted by C1q on cells of both innate and adaptive immunity^50^,^51^,^52^,^53^ as well as on specialized cells localized in tissues such as trophoblasts in placental decidua and microglial cells in the central nervous system^39^,^54^. C1q levels have been reported to be higher in aging brain and to contribute to age-related cognitive decline^55^. In pregnancy C1q has been implicated in tissue remodelling in maternal decidua required for successful embryo implantation^56^. In this report, we describe for the first time a critical role for C1q in tumour development. Compared with WT mice, C1q-deficient mice bearing a syngeneic B16/F10 melanoma had a markedly slower tumour growth with increased survival. Failure to detect significant differences in tumour growth and survival between *C3*^−/−^ and WT mice ruled out the possibility that the cancer-promoting effect of C1q was dependent on C activation. This conclusion was further supported by the analysis of the tumour mass in WT mice that revealed marked deposition of C1q on vascular endothelium and stroma in the absence of C4, while staining for C3 was almost exclusively restricted to CD68^+^ cells and was nearly undetectable when using an antibody detecting C3 activation fragments. C5-deficient mice were also included in our study as a control group because previous reports have shown that C5a, generated through local C activation, promotes tumour growth by recruiting MDSCs that can inhibit T-cell-mediated anti-tumour responses^30^,^57^. The finding that the melanoma cells grew in *C5*^−/−^ mice in a manner similar to that observed in *C3*^−/−^ and WT mice ruled out the contribution of C activation products, and more specifically of C5a, to tumour progression under our experimental conditions. Furthermore, a potential effect of C1q on the anti-tumour immune response was excluded by the analysis of the peri-tumoral cell infiltrates that revealed no differences in the percentage or phenotype of effector T cells, macrophages and MDSCs between *C1qa*^−/−^ and WT mice. None of the immunological parameters analysed showed a statistical significant difference between the two experimental groups. Together the *ex vivo* and *in vivo* data pointed to a direct contribution of C1q to tumour growth independent of C activation and raised the question of whether locally produced C1q was responsible for its cancer-promoting effect.

It is well-known that BM-derived cells are the main source of circulating C1q^41^. However, several other cell types can also synthesize C1q and their contribution to the C1q-mediated tumour-promoting effect was studied in BM transplanted mice. Surprisingly, BM cells from WT mice, despite their ability to reconstitute serum C1q levels in *C1qa*^−/−^ animals had only a negligible effect on tumour growth in these mice suggesting that non-BM-derived cells were the relevant source of C1q needed for its tumour-promoting activity. Included in this group of cells, in addition to stromal cells, are endothelial cells that displayed strong C1q staining in tumour developed in WT mice. Serum was an unlikely source for the C1q bound to endothelial cells because tumour endothelial cells from WT mice reconstituted with C1q-deficient BM cells still expressed C1q despite its nearly undetectable level in circulation. A potential explanation for the C1q expression on the surface of endothelial cells is that these cells synthesize and secrete C1q in response to developing tumour. Although we did not specifically address this point, there is evidence from our previous studies that endothelial cells may acquire the ability to produce C1q at some tissue sites and in certain pathophysiologic conditions like decidual endothelial cells in pregnancy^49^ and dermal microvascular endothelial cells in wound healing^40^. Consistent with this notion we found an increased number of vessels both within the tumour mass and in the peri-tumoral area. This finding extends to tumour, our previous observations that C1q exhibits a pro-angiogenic activity in wound healing^40^. As melanoma is considered a highly angiogenic tumour that exploits newly formed vessels to receive oxygen and nutrients and to establish metastasis^58^,^59^, it was not surprising to see larger metastatic areas in tumour-bearing WT mice compared with *C1qa*^−/−^ mice. Tumour cells need to proliferate to expand and C1q was also able to promote the growth of the melanoma cells *in vitro*. Furthermore, as already seen with trophoblasts^39^, melanoma cells bound to C1q appeared to maintain a round morphology and this facilitates their movement towards surrounding stromal areas and the subsequent dissemination. Therefore our findings suggest that, by interacting with proteins of the extracellular matrix like FN, C1q offers an anchor to tumour cells for their initial seeding. The pro-adhesive effect of C1q does not seem to be restricted to the primary tumour, but can also be seen at metastatic sites as suggested by its presence at lung metastases of colon carcinoma. Among the different C1q receptors/binding molecules reported in the literature, the receptor for the globular portion of C1q (gC1qR), which was previously found to be expressed on trophoblasts and to be capable of mediating C1q-induced cell adhesion and migration^39^, was considered the most likely candidate. Unfortunately, staining of the murine B16/F10 melanoma cells with the mAb 60.11 against human gC1qR, that has been used to visualize the receptor on human cancer cells^60^, failed to detect any gC1qR expression. The negative staining could be due to a poor cross-reactivity of the antibody on murine cells, but we cannot exclude that other molecule(s) may be involved. Further investigations will be required to define the underlying mechanisms of the C1q-mediated effects.

In conclusion, evidence collected from human tumours revealed a persistent presence of C1q, mainly localized in the stroma and on vascular endothelium, in the absence of C4. Consistent with this, in a syngeneic melanoma model the lack of C1q, but not of C3 or C4, reduced the tumour growth and invasion. This tumour-promoting C1q-mediated effect was found to be independent of the circulating C1q levels indicating that non-BM-derived C1q might help tumour progression by facilitating cancer cell seeding and promoting angiogenesis. Our finding that C1q produced in the tumour microenvironment favours cancer development is supported by a recent observation by Winslow *et al.*^61^ that the genes for C1q A-, B- and C-chains were highly expressed in the stroma of breast cancers with poor prognosis.

## Methods

### Human tissues

Tumour tissue samples were obtained from the archives of the Department of Human Pathology, University of Palermo, Italy. Thirty invasive malignant neoplasm specimens were selected including six cases of colon adenocarcinoma, six cases of melanoma, six cases of lung adenocarcinoma, six cases of breast adenocarcinoma and six cases of pancreatic adenocarcinoma. The study was approved by the Institutional review board of the University of Palermo. A specific informed consent was not requested at the time of tissue sample collection for the immunohistochemical analysis of archival tissue sections provided that the patients were not identified and genetic analysis was not performed.

### Mice

C57BL/6 WT mice were purchased from Harlan Laboratories. Mice deficient in C components C1q, C3 or C5 (respectively *C1qa*^−/−^, *C3*^−/−^ and *C5*^−/−^) were generated as described previously^62^,^63^,^64^. All strains were backcrossed onto the C57BL/6 (B6) genetic background for more than 10 generations. All animals were handled in accordance with the institutional guidelines and in compliance with the European (86/609/EEC) and the Italian (D.L.116/92) laws. The Institutional Animal Care Committee of the University of Trieste and the UK Home Office approved the procedures.

### *In vivo* tumour models

B16/F10 melanoma (CRL-6475) and Lewis lung carcinoma (LLC-1, CRL-1642) cell lines were purchased from the American Tissue Culture Collection (Manassas,VA) and confirmed to be mycoplasma free. The cells were maintained in Minimum Essential Medium (Euroclone) supplemented with 10% (v/v) foetal bovine serum, 1% (v/v) penicillin–streptomycin solution, 2 mM L-glutamine, 100 μM non-essential amino acids, 1 mM sodium pyruvate and 10 mM HEPES (all from Life Technologies, Paisley, UK) in a humidified 5% CO_2_ atmosphere at 37 °C. C57BL/6 WT, C57BL/6.*C1qa*^−/−^, C57BL/6.*C3*^−/−^ or C57BL/6.*C5*^−/−^ were injected intramuscularly into the left flank of the mice with viable B16/F10 cells (2 × 10^6^) and tumour development was monitored daily. Both male and female mice (8–12 weeks of age) were used. In each experiment the mice were sex- and age-matched. Tumour size was measured every other day for the first week and then daily with calliper by determining two orthogonal axis, and the volume was calculated using the following formula: (*π*/6)·*α*^2^·*β*, with *α* the shorter and *β* the longer axis^65^. Measurements were performed by a blinded researcher. According to our protocol (D.L.116/92), mice were culled before they developed any signs of distress. When the animals were killed, tumours, spleens and lungs were collected for analyses.

### BM transplantation and assessment of C1q levels

Eight-week-old female mice were irradiated at 8 Gy using a ^137^Cs γ-ray source and reconstituted with 10^7^ BM cells from sex-matched WT or *C1qa*^−/−^ donors. Mice were kept under sterile conditions for the first 2 months after BMT. The successful reconstitution of the haematopoietic lineages after BMT was monitored by flow cytometry and by measuring C1q levels in serum. Two months after the BMT mice were injected intramuscularly with 2 × 10^6^ viable B16/F10 cells and tumour growth monitored up to day 14. Serum C1q levels were measured by ELISA as previously described^41^,^42^. Briefly, microtitre plates were coated with 1 μg ml^−1^ of anti-mouse C1q Ab (clone Rm C7H8; Connex) and blocked with 5% milk in PBS. Serum samples were diluted appropriately in PBS–2%, BSA–0.05% and Tween 20–0.02% NaN_3_. Bound C1q was detected with a biotinylated goat anti-mouse C1q antibody (provided by F. Petry, Mainz, Germany, 1:10,000 dilution) followed by a streptavidin–alkaline phosphatase conjugate (BD Bioscience, San Diego, 1:5,000 dilution) and revealed with *p*-nitrophenyl phosphate alkaline phosphates substrate (Sigma-Aldrich). Optical density (OD) was measured at 405 nm. The results were expressed in μg ml^−1^, referring to a standard curve derived from a known concentration of purified human C1q. C1q-deficient mouse serum was included as a negative control.

### Immunohistochemical analysis

Immunohistochemistry was performed using a polymer detection method. Tissue samples were fixed in 10% buffered formalin and paraffin embedded.

Four-micrometer-thick tissue sections were deparaffinized and rehydrated. The antigen unmasking technique was performed using Novocastra Epitope Retrieval Solutions pH 6, pH 9 and pH 8 in a PT Link Dako pre-treatment module at 98 °C for 30 min. The sections were then brought to room temperature and washed in PBS. After neutralization of the endogenous peroxidase with 3% H_2_O_2_ and Fc blocking by a specific protein block (Novocastra, UK), the samples were incubated overnight at +4 C° with the primary antibodies including rabbit polyclonal anti-human C1q (Dako, Code A0136, 1/500 dilution), goat polyclonal anti-human C1s (Quidel, Catalogue number A302, 1/100 dilution), rabbit polyclonal anti-human C3d (Catalogue number 403A-76, 1/100 dilution) and anti-human C4d (Catalogue number 404A-16, 1/100 dilution) both from Cell Marque Sigma-Aldrich, mouse monoclonal anti-human CD31 (PECAM-1) (clone 1 A10, 1/50 dilution) and anti-human CD34 (clone QBEnd/10, 1/100 dilution) both from Leica Biosystems (Newcastle, UK). IgG from normal mouse, rabbit and goat sera were used as negative controls. Staining was revealed by the horseradish peroxidase (HRP) polymer detection kit (Novocastra, Code RE7280-K) and AEC (3-amino-9-ethylcarbazole) substrate-chromogen. Primary antibodies made in goat were detected with HRP-conjugated rabbit anti-goat IgG Fc (Life Technologies Corporation, Catalogue number A16148, 1/500 dilution) after incubation for 1 h at room temperature. The sections were counterstained with Harris haematoxylin (Novocastra) and analysed under a Leica DMD108 optical digital microscope (Leica Microsystems, Germany).

For double-marker immunohistochemistry, the sections underwent two sequential rounds of single-marker immunostaining. The tissue samples were incubated first with the mouse monoclonal anti-human CD34 clone (1/100 dilution) revealed by the HRP polymer detection kit and AEC substrate chromogen. After Fc blocking, the sections were then incubated with the rabbit anti-human C1q (1/500 dilution) revealed by LSAB+ System-AP (Dako Code K0689) and BCIP/NBT substrate chromogen (Dako Cytomation).

### Immunofluorescence analysis

Double-marker immunofluorescence stainings on paraffin-embedded human tissues processed as described above for the immunohistochemical analysis were performed using the following primary and secondary antibodies: rabbit anti-human C1q 1/500; mouse monoclonal anti-human multi-cytokeratin (Clone AE1/AE3, 1/100, pH 6, Leica Biosystems Newcastle Ltd); mouse monoclonal anti-human Vimentin (Ventana, Clone V9, ID 790–2917, ready to use); mouse monoclonal anti-human anti-melanosoma (Ventana, Clone HMB45, ID 790–4336, ready to use); mouse monoclonal anti-human CD45 (LCA) (Ventana, Clone RP2/18, ID 760-2505, ready to use). After Fc blocking, bound antibodies were revealed by the following fluorochrome-conjugated secondary antibodies: Alexa Fluor 568 conjugated goat anti-Rabbit IgG (H+L) (Catalogue number A11011 1/300 dilution) and Alexa Fluor 488-conjugated goat anti-mouse IgG (H+L) (Catalogue number A11001, 1/350 dilution) both from Invitrogen Molecular Probes (Carlsbad, CA). The slides were counterstained with DAPI Nucleic Acid Stain (Invitrogen Molecular Probes, Catalogue number D1306). All the sections were analysed under a Leica DM2000 optical microscope (Leica Microsystems) and microphotographs were collected using a Leica DFC320 digital camera (Leica).

Seven-micrometer-thick tissue sections of snap-frozen tumour masses from WT and *C1qa*^−/−^ mice at the indicated time points, embedded in OCT medium (Diagnostic Division; Miles Inc), were stained for immunofluorescence analysis with the following primary antibodies: rat monoclonal anti-mouse C1q (clone 7H8; HyCult, 1/50 dilution) and rabbit polyclonal anti-mouse C1q (kindly provided by Prof M. Daha, Leiden, The Netherlands, 1/200 dilution); rat monoclonal anti-mouse C4 (HyCult, clone 16D2, 1/50 dilution), FITC-conjugated F(ab′)2 fragment goat anti-mouse C3 (Cappel, Catalogue number 55510, 1/500 dilution), rat monoclonal anti-mouse C3b/iC3b/C3c (Hycult, clone 2/11, 1/50 dilution). Rabbit antibody to human von Willebrand Factor (vWF) cross-reacting with the murine counterpart (Dako, code number A0082; 1/400 dilution) and rat monoclonal anti-mouse CD68 (AbD Serotec, clone FA-11, 1/200 dilution) were used for double-staining analysis. In some cases, the sections were double stained with rabbit anti-mouse C1q (1/200 dilution) and goat anti-mouse C3 (1/500 dilution) antibodies) or with goat anti-mouse C3 and rat anti-mouse C3b/iC3b/C3c (1/50 dilution) antibodies. After Fc blocking, bound antibodies were revealed by the following fluorochrome-conjugated secondary antibodies: Cy3-coniugated F(ab')2 fragment goat anti-rabbit IgG (Catalogue number 111-166-045, 1/200 dilution) and Alexa Fluor 488 goat anti-rat IgG (Catalogue number 112-545-167, 1/200 dilution) both from Jackson ImmunoResearch. The sections were incubated with DAPI (Sigma-Aldrich, Catalogue D 9542, 1/1,000 dilution) to counterstain cell nuclei. The slides were mounted with the Mowiol-based antifading medium (Sigma-Aldrich, Catalogue number 9002-89-5) and analysed in a blinded manners. Images were acquired with fluorescence microscope Leica DM2000 equipped with Leica DFC420 camera.

### Confocal analysis

B16/F10 cells (3 × 10^5^) were plated at 37 °C on eight-chamber culture slides (BD Biosciences) coated with C1q (20 μg ml^−1^), FN (20 μg ml^−1^), FN+C1q (20 μg ml^−1^ each) or poly-lysine (PolyLys; 70 μg ml^−1^) and left to adhere for 30 min. The cells were fixed and permeabilized with the FIX & PERM Cell Permeabilization Kit (Società Italiana Chimici, Italy), stained with N-(7-nitrobenz-2-oxa-1,3-diazol-4-y)-conjugated phallacidin (NBD-phallacidin) (Molecular Probes, Invitrogen 1:50 dilution) and mouse monoclonal anti-paxillin (Merck-Millipore, clone 5H11, 1:100 dilution) followed by Cy3-conjugated F(ab′)_2_ goat anti-mouse IgG (Jackson ImmunoResearch Catalogue number 115-166-062, 1:300 dilution) as previously described^39^,^66^. Images were acquired with the Leica TCS SP2 confocal system (Leica Microsystems) using the Leica Confocal Software and a 633 fluorescence objective on a Leica DM IRE2 microscope (Leica Microsystems) or with a Nikon C1Si confocal system, using the Nikon EZ-C1 Confocal Software and a 633 fluorescence objective on a Nikon TE2000-U inverted microscope (Nikon, Melville, NY).

### Evaluation of angiogenesis and metastases

The microvascular density was evaluated on CD31-immunostained sections from mouse tumour samples by counting the number of vessels out of 10 microscopic fields at × 200 magnification and averaging the counts. The number of lung parenchymal metastases was evaluated on haematoxylin and eosin-stained sections and the overall metastatic burden was assessed as the sum of the areas of the metastatic foci in the evaluated sections. Measurements were performed using a Leica DMD108 digital microscope. The analyses were carried out in a blinded manner.

### Flow cytometry

Single cell suspensions were stained using standard protocols in the presence of a saturating concentration of anti-mouse CD16/CD32 (2.4G2) mAb. The following antibodies were used: anti-CD4 (clone RM4-5, 1:100 dilution), anti-CD8a (clone53-6.7, 1:100 dilution), anti-CD11b (clone M1/70, 1:200 dilution), anti-CD25 (clone eBio7D4, 1:100 dilution), anti-CD44 (clone IMF, 1:200 dilution), anti-CD45 (clone 30-F11, 1:200 dilution), anti-CD62L (clone MEL-14, 1:100 dilution), anti-CD69 (clone H1.2F3, 1:100 dilution), anti-Foxp3 (clone FJK-16 s, 1:100 dilution) and Gr1 (clone RB6-8C5, 1:100 dilution). For intracellular staining, cells were re-stimulated with 10 ng ml^−1^ of Phorbol 12-Myristate 13-Acetate (PMA) (Sigma) and 250 ng ml^−1^ of Ionomycin (Calbiochem) in the presence of GolgiStop (BD Pharmingen) for 4 h. Cells were then permeabilized with Cytofix/Cytoperm (BD Biosciences) before being stained for Granzyme B (clone NGZM, 1:100 dilution), IFN-γ (clone R4-6A2, 1:100 dilution) and IL-2 (clone ES6-5H4, 1:100 dilution). Antibodies were all purchased from eBioscience (Life Technologies). Data were acquired using a FACSVerse (Becton Dickinson, Mountain View, CA) and analysed using FlowJo software, version 7.6 (TreeStar, Ashland, OR).

### Adhesion assay

The adhesion assay was performed as previously reported^39^. Briefly, 1 × 10^5^ B16/F10 cells per 100 μl RPMI 1640 (Life technologies) containing 0.1% (v/v) BSA (Sigma-Aldrich, Catalogue number A 2058), labelled with the fluorescent dye FAST DiI (1:100 dilution), were added to a 96-well plate (Iwaki, Bibby Scientific Italia, Italy) for 35 min at 37 °C in an air/CO_2_ incubator. The wells were coated with BSA, C1q (Sigma-Aldrich Catalogue number C1740), and FN (Roche Life Science, Catalogue number 11051407001) used at the concentration of 20 μg ml^−1^ in pH>9 sodium bicarbonate-buffered medium (100 mM). PolyLys (Sigma-Aldrich, Catalogue number P7280) was used as a negative control. After incubation, the unbound cells were removed and the number of adherent cells was counted with Infinite200 (absorbance 544 nm, emission 590 nm) (TECAN Italia, Italy) and referred to a calibration curve established with an increasing number of labelled cells.

### Cell proliferation

B16/F10 cells were serum-starved for 24 h, suspended in serum-free medium containing 0.1% BSA and seeded in 24 well plates (Corning) at 3 × 10^5^ cells per well. Prior to cell seeding, the wells were coated with PolyLys (70 μg ml^−1^), C1q, FN or C1q and FN together as described above. After 18 h, the supernatant was removed, the cells were lysed with ZAP-OGLOBIN II Lytic Reagent (Beckman Coulter) and counted with Coulter Particle Counter Z1 (Beckman Coulter). *In vivo* proliferation studies were performed by injecting 2 mg of BrdU (5-bromo-2′-deoxyuridine; eBioscience) i.p. 4 days after the intramuscular injection of B16/F10 (2 × 10^6^) cells. Mice were killed 3 h after the BrdU administration. Tumour cells were harvested and stained for BrdU incorporation according to manufacturer's instructions (BrdU Staining Kit for Flow Cytometry FITC, eBioscience). Tumour cells from non-BrdU-injected mice were used as negative controls for gating the BrdU^+^ cells.

### Apoptosis

Serum-starved B16/F10 were suspended in serum-free medium containing 0.1% BSA and seeded at 2 × 10^4^ cells per well on plates pre-coated with PolyLys, C1q, FN and the mixture of C1q and FN as reported above. The cells were left to adhere for 1 h at 37 °C and subsequently incubated with 500 μM H_2_O_2_ for 6 h followed by the addition of 5 μM of CellEvent Caspase-3/7 Green Detection Reagent (Life Technologies), a fluorogenic substrate for activated caspases 3 and 7. The reagent consists of a non-fluorescent four amino acid peptide (DEVD) conjugated to a nucleic acid-binding dye. After activation of caspase-3 or caspase-7 in apoptotic cells, the DEVD peptide is cleaved, enabling the dye to bind to DNA and to produce a bright fluorogenic response with an absorption/emission maxima of ∼502/530 nm. The fluorescence data were acquired with TECAN Infinite200.

### Migration assay

The migration assay was performed as previously reported^39^ using FAST DiI-labelled (1:100 dilution). B16/F10 cells (2 × 10^5^ cells) were resuspended in RPMI 1640 with 0.1% (v/v) BSA and added to the upper chamber of a transwell system. The cells were allowed to migrate through HTS FluoroBlok systems with polycarbonate membranes of 8-μm pore size (Becton Dickinson, Falcon, Italy) coated on the upper side with BSA, C1q and FN used at the concentration of 20 μg ml^−1^ in pH>9 sodium bicarbonate-buffered medium (100 mM). Double-coated wells were prepared as described above. After 18 h, the number of cells migrated to the lower side of the insert was evaluated as outlined above for the adherent cells.

### ROS and RNS

Tumour-infiltrating leucocytes were resuspended in DMEM and incubated with 2 μM of oxidation-sensitive dye H_2_DCFDA (2′-7′-dichloro dihydrofluorescein diacetate; Molecular Probes) at 37 °C for 15 min. Cells were then washed with PBS and stained with antibodies against CD45, CD11b and Gr1. The fluorescent intensity of DCF, that correlates with reactive oxygen species (ROS) and reactive nitrogen species (RNS) production, within the MDSCs (CD45^+^CD11b^+^Gr1^+^ population) was measured by flow cytometry according to manufacturer's instructions (Molecular Probes). The amount of ROS and RNS was expressed as median fluorescence intensity of the gated populations.

### MDSC-mediated T-cell suppression assay

MDSCs were isolated from tumours from WT and *C1qa*^−/−^ mice using Gr1 microbeads (Miltenyi Biotec). Lymph node cells (1 × 10^5^) from naïve *C1qa*^−/−^ mice were labelled with CFSE (5-(and-6)-carboxyfluorescein succinimidyl ester; Molecular Probes) and stimulated with CD3/CD28 dynabeads (Invitrogen) at 1:1 ratio (beads/cells) in the presence or absence of WT or *C1qa*^−/−^ MDSCs (5 × 10^4^). T-cell proliferation was assessed by flow cytometry measuring the CFSE dilution after 3 days of co-culture. Unstimulated CFSE-labelled lymphocytes were used as basal proliferation control.

### Statistical analysis

Data from *in vivo* mouse models were analysed using two-way analysis of variance (ANOVA), Tukey–Kramer test, and *in vitro* experiments with unpaired two-tailed Student *t*-test or one-way ANOVA with Bonferroni corrections. Results were expressed as mean±s.e.m. Non-parametric data were assessed by Mann–Whitney *U*-tests and the results were expressed as median and interquartile range. *P* values of <0.05 were considered significant. All statistical analyses were performed using Prism 6 software (GraphPad Software Inc, La Jolla, CA).

## Additional information

**How to cite this article:** Bulla, R. *et al.* C1q acts in the tumour microenvironment as a cancer-promoting factor independently of complement activation. *Nat. Commun.* 7:10346 doi: 10.1038/ncomms10346 (2016).

## Supplementary Material

Supplementary Information Supplementary Figures 1-7.
