# Supplementary material for: C1q acts in the tumour microenvironment as a cancer-promoting factor independently of complement activation
Source: Nat Commun. 2016 Feb 1;7:10346. doi: 10.1038/ncomms10346 (PMC4740357; doi:10.1038/ncomms10346)
Supplement: Supplementary Information — Supplementary Figures 1-7. [file ncomms10346-s1.pdf]

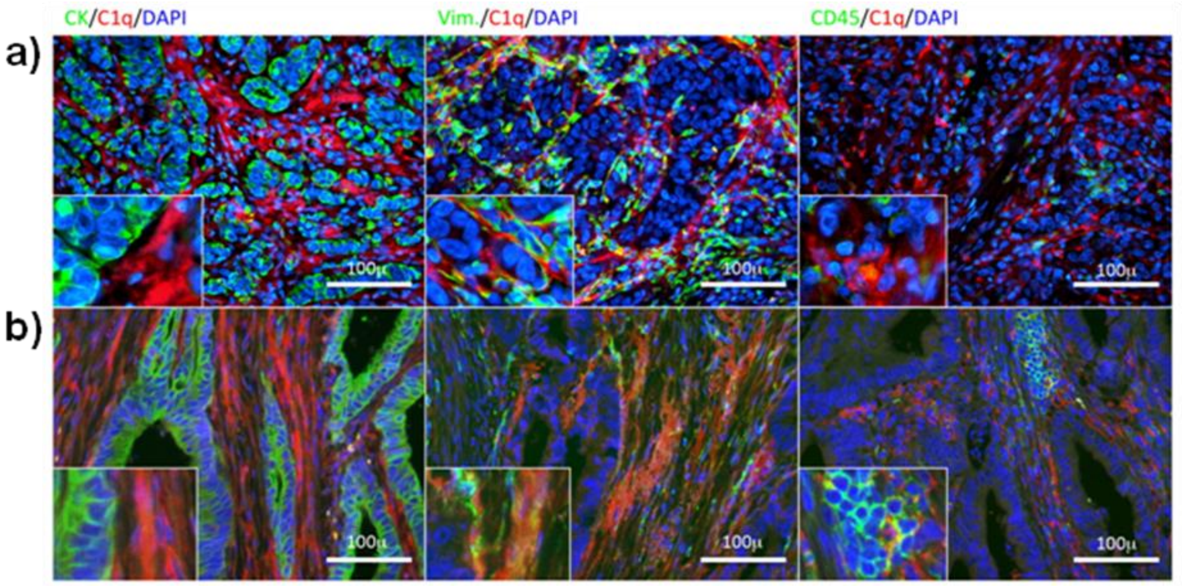

Supplementary Figure 1. Double-staining immunofluorescence analysis of invasive colon and breast cancers. Specimens from invasive ductal breast carcinoma (a) and colon adenocarcinoma (b) were staining for epithelial (cytokeratin, upper and lower left panels, green signal) or mesenchymal (vimentin, upper and lower middle panels, green signal) or immune (CD45, upper and lower right panels, green signal) cell markers and C1q (red signal). C1q expression was not observed in the neoplastic cells identified by the cytokeratin staining, whilst it was detectable in vimentin-expressing mesenchymal elements and in some CD45-positive immune cells within the tumor-associated stromal microenvironment. Scale bars, 100μm.

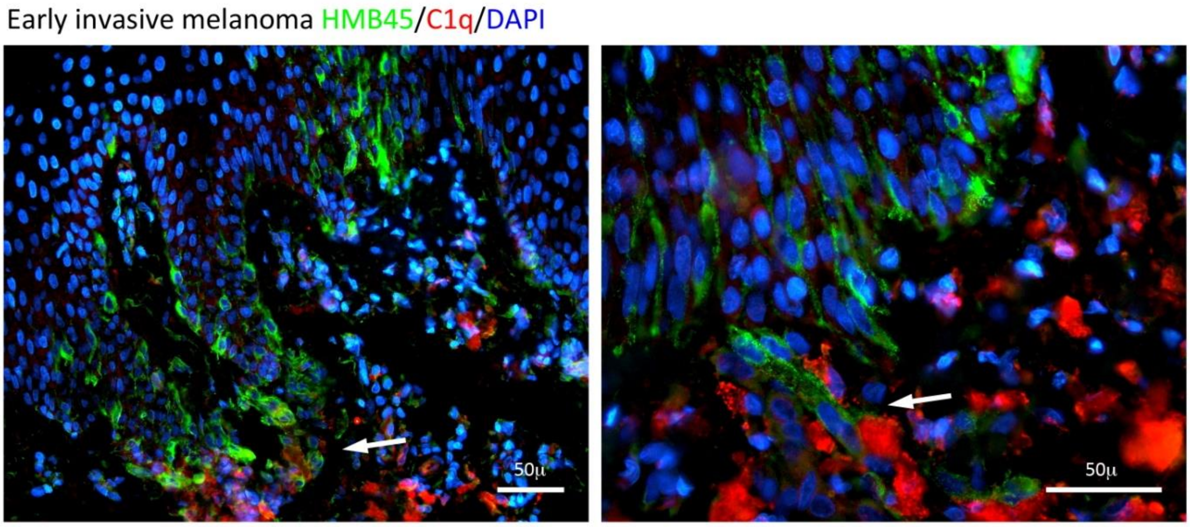

Supplementary Figure 2. Double-marker immunofluorescence analysis of early invasive melanoma. The malignant melanocytic marker HMB-45 (green signal) was used to identify melanoma infiltrating cells. C1q (red signal) is expressed by non-tumoral cells of the dermal-epidermal junction at the edge of cancer invasion (arrows). Scale bars, 50μm.

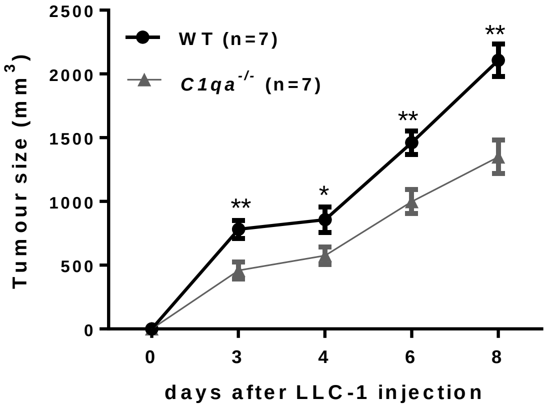

Supplementary Figure 3. Evaluation of tumour size. 1x10^6 Lewis lung carcinoma cells (LLC-1) were injected intramuscularly in WT (circle) and *C1qa*$^{-/-}$ (triangle) mice. Tumour mass measured at different days after tumour cell injection. Data are shown as mean ± SEM. *p <0.05, **p <0.01. Student *t*-test at each time point.

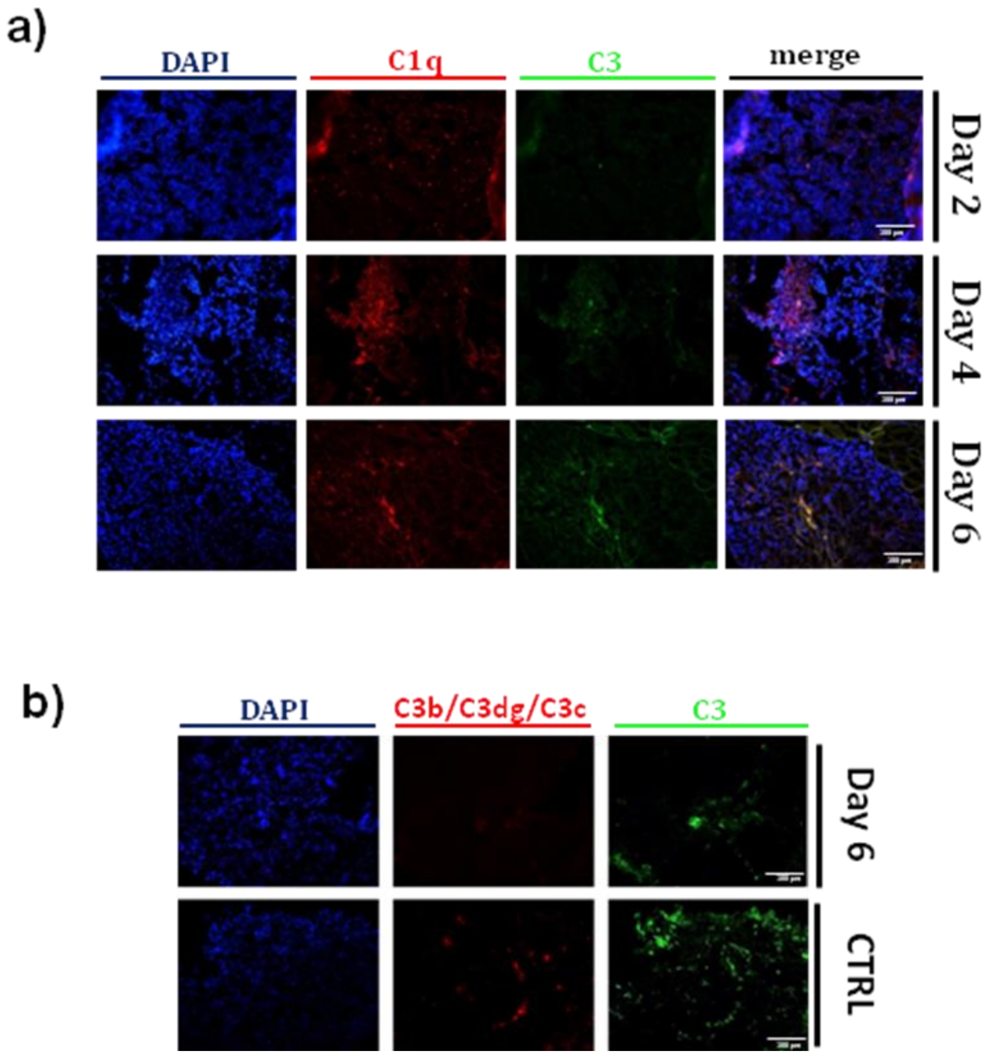

Supplementary Figure 4. a) Kinetic analysis of C1q and C3 deposition on developing melanoma tumour. Expression of C1q (red) and C3 (green) in tumour tissue collected from WT mice at different days after B16/F10 injection. Scale bars, 100 µm. b) Analysis of C3 using two different antibodies that recognize either intact (green) or cleaved C3 fragments (red). A lymphoma model developed in SCID mice and tagged with a complement-fixing antibody served as positive control (ctrl). Scale bars, 100 µm.

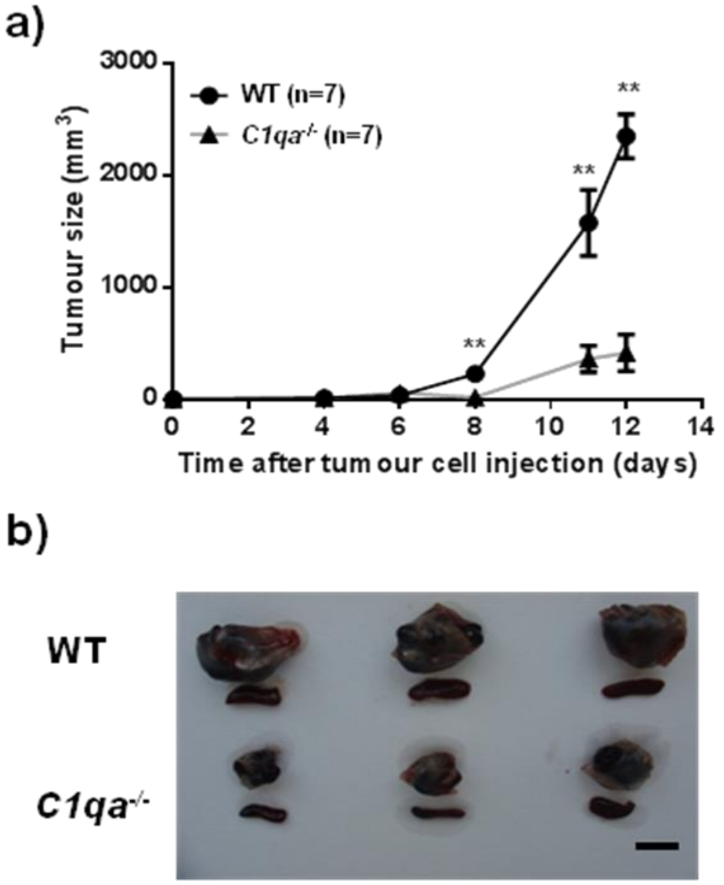

Supplementary Figure 5. Evaluation of melanoma tumour size in WT and C1q-deficient mice. a) Tumour mass of *C1qa*$^{-/-}$ mice (triangle) and WT mice (circle) measured at different days after tumour cell injection. Data are shown as mean ± SEM. **$p<0.01$, Student *t*-test at each time point. Representative data of 4 independent experiments. b) Representative imagines of the tumours and spleens at day 12. Scale bar, 1 cm.

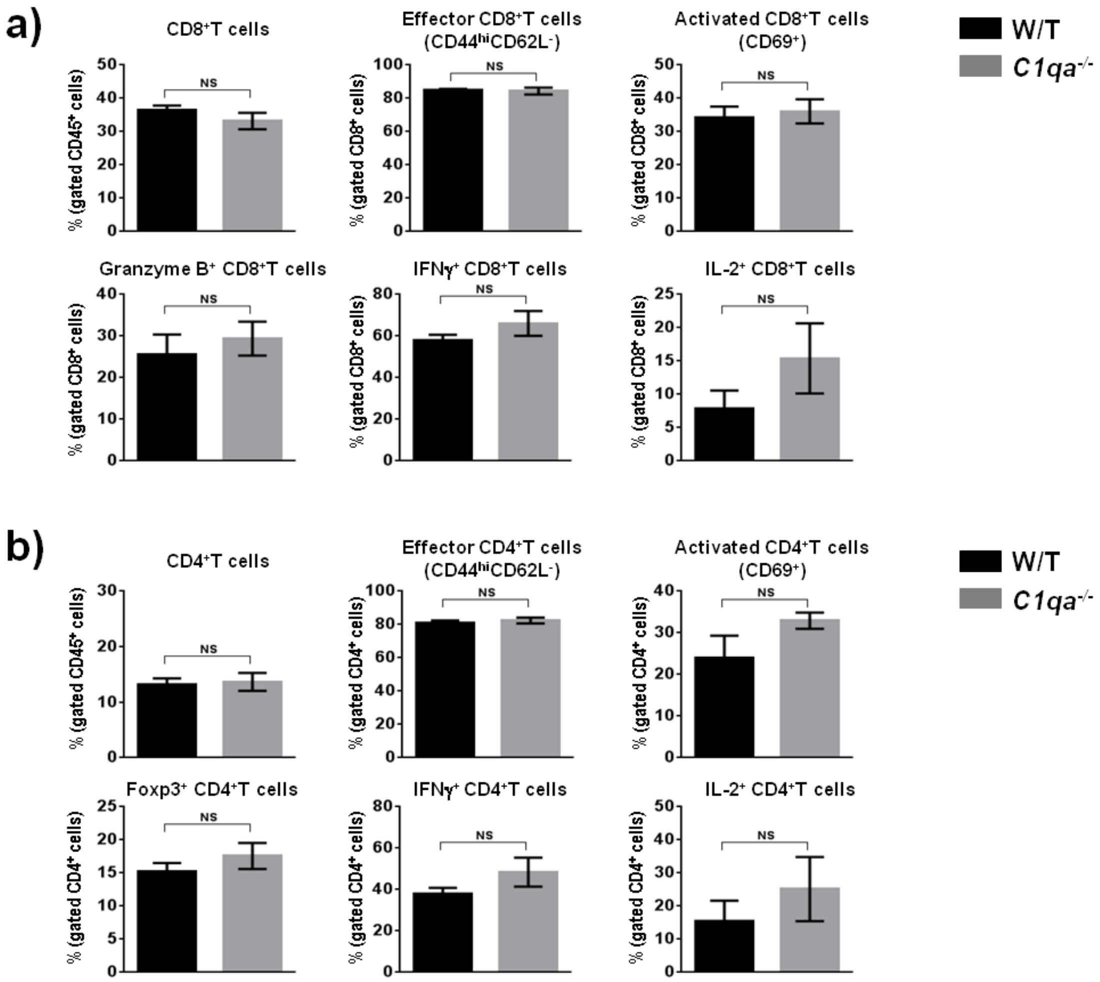

Supplementary Figure 6. Phenotypic analysis of tumour-infiltrating immune cells. Graphs showing the percentage and phenotype of tumour-infiltrating CD8$^+$ (a) and CD4$^+$ (b) T cells within the tumour mass 12 days after the B16/F10 injection. a) Frequency of CD8$^+$ T cells expressing granzyme B, IFN-g and IL-2 are shown. b) Percentage of CD4$^+$ T cells expressing Foxp3 (regulatory T cells), IFN-g and IL-2 isolated from tumours of WT and *C1qa*$^{-/-}$ mice. Analysis by flow cytometry. Data are mean ±SEM (n=4-5/group) and are representative of three experiments. NS = not significant, statistical analysis by Student *t-test*.

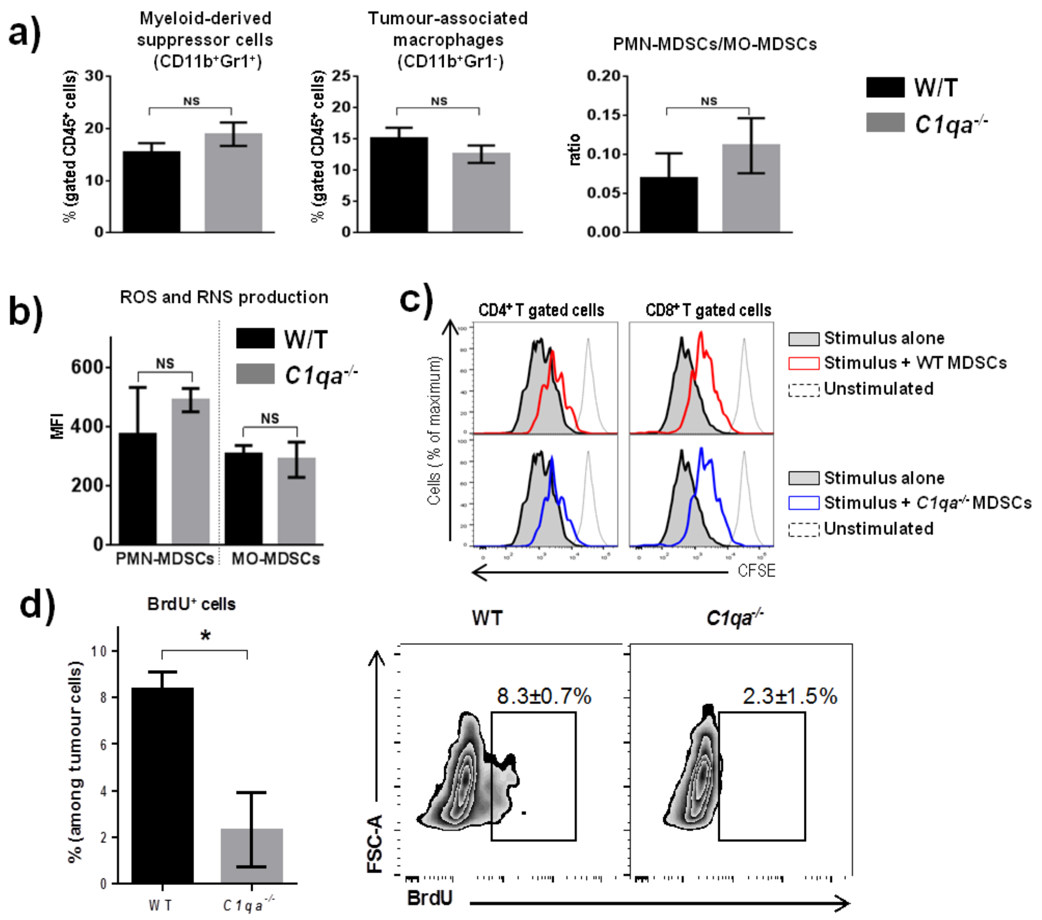

Supplementary Figure 7. Analysis of myeloid-derived suppressor cell populations. Graphs showing the percentage of myeloid-derived suppressor cells (MDSCs) and tumour-associated macrophages within the tumour mass 12 days after the B16/F10 injection. a) Percentage of MDSCs and tumour-associated macrophages among CD45^{+} tumour-infiltrating cells. Ratio of PMN-MDSCs (CD11b^{hi}Gr1^{+}) to MO-MDSCs (CD11b^{+}Gr1^{+}) within the total tumour MDSC population. PMN-MDSCs were defined as having higher expression of CD11b and Gr1 compared to MO-MDSCs. b) Quantification of reactive oxygen species (ROS) and reactive nitrogen species (RNS) production by PMN-MDSCs and MO-MDSCs isolated from tumours of WT and *C1qa*$^{-/-}$ mice. MFI = median fluorescence intensity. c) Proliferation of CD4^{+} or CD8^{+} T cells, isolated from non-tumour bearing *C1qa*$^{-/-}$ mice, stimulated with CD3/CD28 beads in the presence (open histogram) or absence (shaded histogram) of MDSCs isolated from tumours from WT (red) and *C1qa*$^{-/-}$ (blue) mice. Unstimulated CFSE-labelled lymphocytes were used as control (dotted line). (a-b) Data are mean ± SEM (n=4-5/group) and are representative of three experiments. (c) MDSCs were isolated from pooled tumour samples of 4 mice per strain. Data are representative of two experiments. d) B16/F10 cells were isolated from WT and *C1qa*$^{-/-}$ mice 4 days B16/F10 post-injection. Tumour cell proliferation, measured by BrdU uptake, was assessed by flow cytometry. Data are shown as mean ± SEM

(n=3). *P<0.05, statistical analysis by Student *t-test.* FSC-A = forward side scatter area, NS = not significant.
